# Academic Detailing Compared with Group Meetings to Change Drug Prescribing for Type 2 Diabetes—A Randomized Controlled Trial

**DOI:** 10.1007/s11606-024-09014-z

**Published:** 2024-09-04

**Authors:** Harald Christian Langaas, Øyvind Salvesen, Roar Dyrkorn, Hege Salvesen Blix, Olav Spigset

**Affiliations:** 1 The Hospital Pharmacy in Trondheim, Edvard Griegs Gt. 10, 7030 Trondheim, Norway; 2 KUPP – The Norwegian Academic Detailing Program, Department of Clinical Pharmacology, St. Olav University Hospital, Trondheim, Norway; 3 Regional Medicines Information and Pharmacovigilance Centre (RELIS), Department of Clinical Pharmacology, St. Olav University Hospital, Trondheim, Norway; 4 Department of Clinical and Molecular Medicine, Faculty of Medicine and Health Sciences, Norwegian University of Science and Technology (NTNU), Trondheim, Norway; 5 Central Norway Pharmaceutical Trust, Trondheim, Norway; 6 Department of Clinical Pharmacology, St Olav University Hospital, Trondheim, Norway; 7 Department of Antibiotic Resistance and Infection Prevention, Norwegian Institute of Public Health, Oslo, Norway; 8 Department of Pharmacy, Faculty of Mathematics and Natural Sciences, University of Oslo, Oslo, Norway

**Keywords:** academic detailing, educational outreach, type 2 diabetes, general practice

## Abstract

**Background:**

Academic detailing (AD) is a one-on-one educational outreach with the goal to improve prescribing. There is insufficient evidence on the difference in impact between AD and group visits to facilitate behavior change among general practitioners (GPs).

**Objective:**

To compare the impact of individual AD visits and group visits conveying the same content on treatment of type 2 diabetes (T2D).

**Design:**

Randomized controlled trial.

**Participants:**

GPs in Central Norway, visited September – November 2018.

**Intervention:**

A total of 210 GPs were randomized and invited to an individual AD visit lasting 20 min; 193 were visited, of whom 146 were included in the analyses. In addition, 293 GPs were randomized and invited to a group meeting lasting 30–45 min; 261 were visited, of whom 188 were included in the analyses. Finally, 167 GPs were randomized and included in a control group. Visits were conducted by trained pharmacists and physicians.

**Main Measures:**

Changes in prescribing of metformin and other T2D drugs after the intervention.

**Key Results:**

The use of metformin increased with 5.9% the year after AD and with 4.9% the year after group meetings, compared to no change (0.0%) in the control group (*p* = 0.006 and *p* = 0.016, respectively). There was no significant difference between the two intervention groups. The only drug group with a statistically significant difference between interventions was insulins, with an increase of 3.2% after AD compared to 19.1% after group visits (*p* < 0.001). For GLP-1 analogues (*p* = 0.031) and T2D drugs in total (*p* = 0.010), we found a significant difference between group intervention and control. Other differences between study groups did not reach statistical significance.

**Conclusions:**

Short educational visits of 20–45 min impact the prescribing of drugs for T2D, either the education is given one-on-one as AD or in a group setting.

**Supplementary Information:**

The online version contains supplementary material available at 10.1007/s11606-024-09014-z.

## INTRODUCTION

Academic detailing (AD) is a documented method for facilitating behavior change in clinicians, where a trained professional (academic detailer) has an interactive meeting about a specific subject with a clinician.^1–3^ The process of AD can be divided into the following four stages: (1) undertaking a literature review to identify and evaluate the evidence; (2) preparing the evidence into a condensed and easily accessible format; (3) choosing and training academic detailers; and finally (4) visiting the prescribers.^4,5^

Originally AD visits were done one-on-one, and the term is usually exclusively used for one-on-one visits, as interaction between prescriber and visitor is a paramount part of AD.^5^ Nevertheless, many group interventions have been published as AD.^6–9^ There are also other substantial variations between interventions published as AD, such as the qualification of the academic detailer, the number of visits per clinician, and the duration of visits.^10^ As the implementation of academic detailing is not uniform, evaluation of its effectiveness is difficult to perform.^4,11–13^

We only identified three previous studies that evaluated differences in impact on drug prescribing between individual and group visits.^14–16^ All three studies were of high methodological quality, being randomized and including a control group. Only one of those studies found statistically significant differences between individual and group visits, with individual visits having bigger impact on prescribing.^14^ Due to the sparse evidence, it is not possible from existing literature to conclude whether one-on-one visits are more effective than group visits in changing prescribing behavior among clinicians.

To further investigate this topic, we designed a randomized controlled study to compare the impact of one-on-one AD visits and group visits conveying the same content. At the time of the study, our academic detailing program (KUPP – The Norwegian Academic Detailing Program) was visiting general practitioners (GPs) on a campaign about the treatment of type 2 diabetes (T2D), and we decided to use this campaign for the study. KUPP is an independent, government-funded initiative using AD to improve prescribing in primary care, run by RELIS (Regional Medicines Information and Pharmacovigilance Centres).^17^ KUPP offers free AD visits to GPs, and has covered various topics, including non-steroid anti-inflammatory drugs (NSAIDs),^18^ opioids,^19^ and antibiotics. The purpose of the campaign on T2D was to support GPs in treating T2D in line with the national treatment guidelines for diabetes, in particular maintaining metformin as the initial drug of choice.^20^ Thus, the aim of the present study was to compare the impact of one-on-one AD visits and group visits on drug prescribing for T2D.

## METHODS

### Intervention

We prepared campaign materials by reviewing the literature on treatment of T2D. The campaign was compliant with the national Norwegian guidelines for treatment of diabetes, updated in April 2018.^21^ For AD visits, we prepared a four-page brochure with advice on treatment choices. For group visits, we prepared a standardized PowerPoint® presentation with the same content and visuals that were used in the brochure. One-on-one visits were conducted in line with the principles of AD, with focus on dialogue and discussions to identify barriers and possible solutions.^5,22^ For one-on-one visits, we asked GPs to set aside a minimum of 20 min.

Group visits were conducted as 30-min presentations for all GPs at the clinic combined, with 15 min for questions and discussion after the presentation if the clinic accepted to set aside 45 min in total. The PowerPoint® presentation consisted of 18 slides.

### Study Setting and Participants

**Figure 1:**
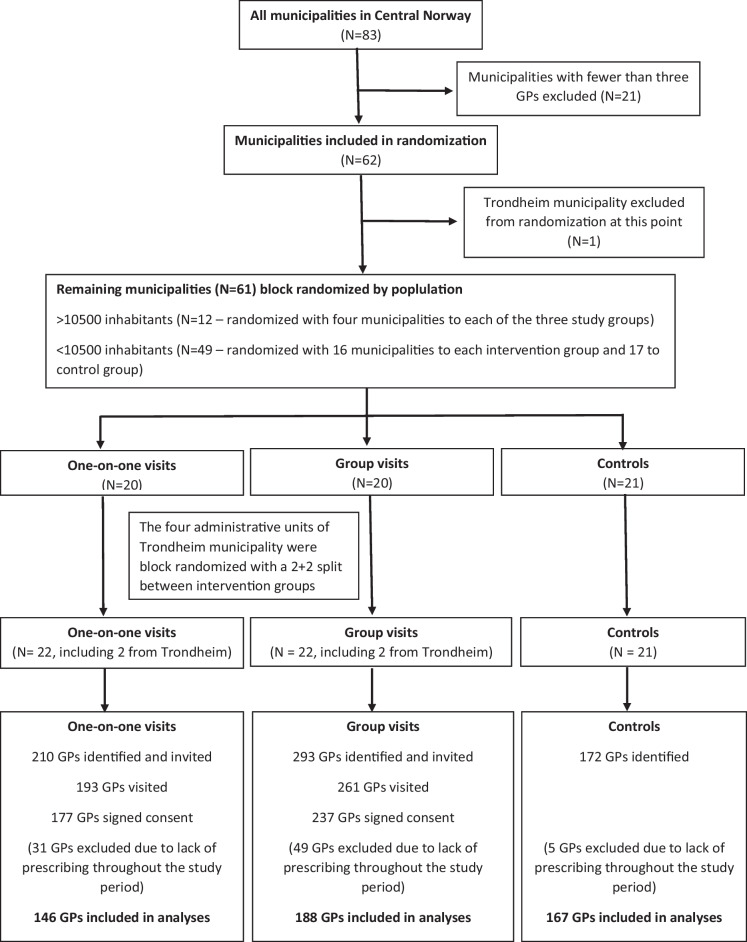
Flowchart showing the inclusion of GPs to the different study groups.

GPs in The Central Norway Health Region were block randomized by municipality to receive a one-on-one visit, a group visit, or no intervention (control). At the time of randomization, The Central Norway Health Region consisted of 83 municipalities. Twenty-one municipalities were excluded because they had fewer than three GPs (our defined minimum for group meeting). The remaining 62 municipalities were stratified by population. The 49 municipalities with less than 10,500 inhabitants, and the 12 municipalities with 10,500–50,000 inhabitants were randomized separately in a 1:1:1 ratio between the three study groups. The largest municipality in the region (the city of Trondheim, 193,000 inhabitants) had more than four times the inhabitants of the second largest municipality, and was excluded from randomization at this stage. As Trondheim consisted of four administrative units, it was not practically feasible to distribute the GPs here in three groups. We therefore decided to randomize the four Trondheim groups to the two intervention groups, thereby ensuring the highest degree of homogeneity between the two intervention groups and also balancing the numbers in them, although to the cost that Trondheim was excluded from the control group. Based on the number of registered GPs in those municipalities, 293 GPs were randomized to a group visit, 210 GPs to a one-to-one visit, and 172 GPs to the control group (Fig. 1). Randomization was done with a sequence generator from the webpage random.org.

All GPs that accepted a visit were asked to sign an informed consent form, allowing us to retrieve anonymous prescription data from The Norwegian Prescription Database (NorPD) 12 months before and after the intervention. Visits were conducted between September and November 2018. GPs received no financial incentive to participate. All visited GPs received an individual anonymous electronic evaluation form (Questback®) with seven questions after the visit.

The study was approved by the Regional Committee for Medical and Health Research Ethics (Ref. No. 12709 REK South-East C).

### Measures

As proxy for prescribing data, dispensing data from the Norwegian Prescription Database (NorPD), a validated population-based data source which contains all dispensing from Norwegian pharmacies, were used.^23–25^ In Norway, most prescriptions for chronic diseases like T2D are valid for repeated dispensing up to 1 year after the date of prescribing. We therefore evaluated changes in dispensing from each GP from 364 days before to 364 days after the intervention. Periods of 364 and not 365 days were chosen to include equal numbers of Saturdays and Sundays in all calculations irrespective of the weekday of intervention, as pharmacy sales on these days clearly differ from average. Since there was no intervention in the control group, intervention dates from the intervention groups were randomly assigned as a fictitious intervention day to each GP in the control group and used in the analyses.

To ensure that all included GPs were actively prescribing T2D drugs throughout the study period, we only included those who met the following criteria: (i) first dispensed prescription of an antidiabetic drug minimum 364 days before the date of intervention, and (ii) last dispensed prescription of an antidiabetic drug to a new patient or of a new antidiabetic drug to an existing patient minimum 364 days after intervention. The same criteria were used for the control group, applying the fictitious intervention date (Fig. 1).

T2D drugs were defined as drugs belonging to Anatomical Therapeutic Chemical (ATC) Classification System group A10B blood glucose lowering drugs, excl. insulins. We included patients being prescribed insulin (ATC group A10A) if they also were dispensed any drug from A10B, thereby excluding patients with type 1 diabetes.^26^ For the analyses, we used the total amount of Defined Daily Doses (DDDs) dispensed for each relevant antidiabetic drug for each prescriber. DDD is defined as the assumed average maintenance dose per day for a drug used for its main indication in adults.^26^ DDDs for fixed combination drugs were recalculated giving a DDD count as if each of the active ingredients had been administered separately.

The following key information was retrieved from NorPD for all prescriptions including T2D drugs: demographic information on the prescribers (gender, age, and speciality) and the patients (gender, age, and municipality), in addition to information related to the dispensed drugs (date of dispensing, ATC code, and amount dispensed in DDD).

### Outcomes

Primary endpoint of the analysis was the change in the amount of prescribing of metformin. Metformin was chosen as primary endpoint as it was the drug of choice in the National guidelines at the time of the intervention.^26^ Secondary endpoints were changes in prescribing of the other groups of T2D drugs and of T2D drugs in total, and results from the evaluations of the visits from the GPs.

### Statistical Analyses

Year-over-year changes in daily dispensed drugs were computed on a daily basis from day 1 (the day after intervention) through day 364 for each of the two groups. These changes were consistent with an identical independent normal distribution for the changes in each group. Year-over-year change in yearly dispensed medication for each group was assessed using the one-sample *t*-test. Pairwise comparisons of year-over-year percentage change in yearly dispersed medication between groups were done using the two-sample *t*-test with Holm-Bonferroni adjustment.

Data preparation and aggregation were done using MySQL version 5.7.34. Statistical analyses on aggregated data were done using R version 3.6.3. The level of significance was taken at 5%.

## RESULTS

Of the 210 GPs randomized and invited to the AD intervention group, 193 were visited. The average time for the one-on-one visits was 25.6 min (range 15 – 45 min) with 92% of visits lasting 20 – 30 min. Of those visited, 177 signed consent and were eligible for inclusion in the study. After excluding GPs that were not actively prescribing antidiabetic drugs throughout the study period, 146 GPs were included in the analyses (Fig. 1).

Of the 293 GPS randomized and invited to group meetings, 261 were visited in a total of 58 meetings. The average number of GPs in each meeting was 4.4 (range 3 – 9). The average time for the meetings was 46.2 min (range 30 – 80 min), with 78% of visits lasting 40 – 60 min. Of those visited, 237 signed consent and were eligible for inclusion in the study. After exclusion of GPs that were not actively prescribing antidiabetic drugs throughout the study period, 188 were included in the analyses (Fig. 1). For the control group, 167 GPs met the inclusion criteria and were included in the analyses (Fig. 1).

Demographic data of the GPs and the patients included are presented in Table 1.

**Table 1:** Demographic Characteristics of the General Practitioners and Patients Included in the Two Intervention Groups and the Control Group

	One-on-one visits	Group visits	Controls
General practitioners			
Number included	146	188	167
Mean age (years)*	45 (SD 10)	46 (SD 11)	47 (SD 12)
Female gender, *n* (%)	61 (41.8%)	85 (45.2%)	66 (39.5%)
Specialist in general practice,* n* (%)	104 (71.2%)	124 (66.0%)	108 (64.7%)
Patients			
Number included	39 663	51 189	35 630
Mean age (years)*	64 (SD 16)	64 (SD 17)	65 (SD 16)
Female gender, *n* (%)	19 947 (50.3%)	25 679 (50.2%)	17 946 (50.4%)

*SD* standard deviation

^*^Age per December 31, 2018

**Figure 2:**
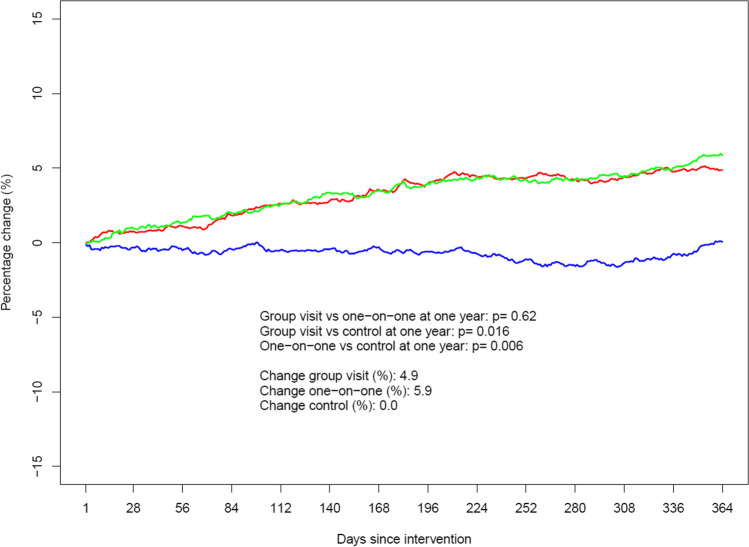
Year-over-year change in yearly dispensed amount for metformin. Fixed combinations with other drugs are included. The green color indicates the one-on-one intervention, red color the group intervention, and blue color the control group. On the y-axis, positive numers denote an increase whereas negative numbers denote a decrease.

For metformin, the total amount prescribed measured in DDD changed by + 5.9% after one-on-one visits and by + 4.9% after group visits the year after the intervention, compared to the year before the intervention (*p* = 0.62). In the control group, there was no change (0.0%). For both interventions, the change was significantly different from the control group (*p* = 0.006 and *p* = 0.016, respectively). The time patterns of the changes in the three study groups are displayed in Fig. 2.

**Figure 3:**
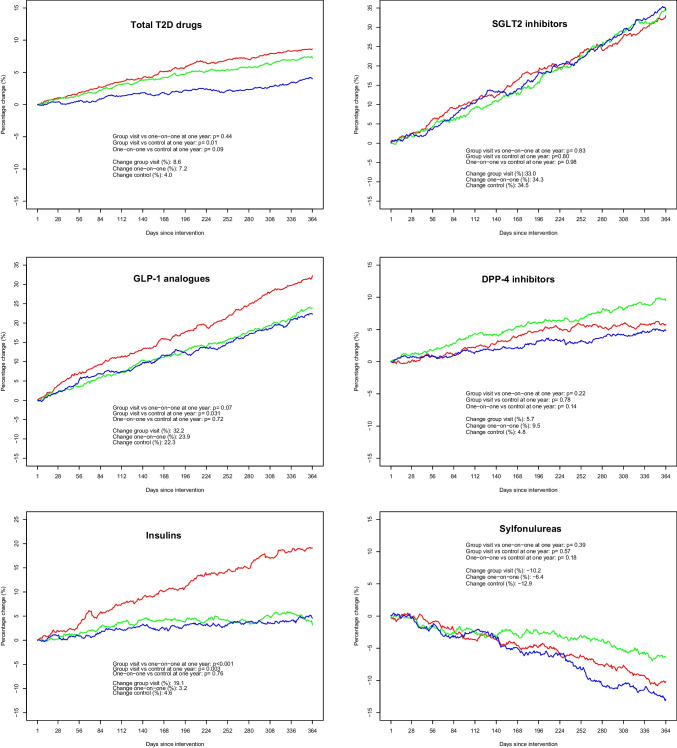
Year-over-year change in the yearly dispensed amount of various drug groups used for type 2 diabetes. The panels from top left represent total T2D drugs (ATC group A10B, plus insulins to patients with T2D), SGLT2 inhibitors (sodium-glucose cotransporter-2 inhibitors), GLP-1 analogues (glucagon-like peptide-1 receptor agonists), DPP-4 inhibitors (dipeptidyl peptidase-4 inhibitors), insulins (ATC group A10A, only to patients also prescribed any drug from ATC group A10B), and sulfonylureas. For all graphs, green color indicates the one-on-one intervention, red color the group intervention, and blue color the control group. On the y-axis, positive numbers denote an increase whereas negative numbers denote a decrease.

For other T2D drugs, results are displayed in Table 2 and Fig. 3. The only drug group with a statistically significant difference between the two intervention groups was insulins, with a change of + 3.2% after one-on-one visits compared to + 19.1% after group visits (*p* < 0.001). The change in the control group was + 4.6% (*p* = 0.003 vs. group intervention, *p* = 0.76 vs. one-on-one intervention). There were also significant differences between group intervention and controls for GLP-1 analogues (+ 32.2% vs. + 23.3%; *p* = 0.031) and for T2D drugs in total (+ 8.6% vs. + 4.0%; *p* = 0.010). No other differences between study groups reached statistical significance.

**Table 2:** Changes in the Amount Prescribed for Subgroups of Antidiabetic Drugs and Antidiabetic Drugs in Total in the Year After the Intervention, Compared to the Year Before the Intervention

Drug/drug group	One-on-one visit	Group visit	*p*-value One-on-one vs. group visit	Controls	*p*-value One-on-one vs. controls	*p*-value Group visit vs. controls
Metformin*	+ 5.9%	+ 4.9%	0.62	0.0%	0.006	0.016
Sulfonylureas	− 6.4%	− 10.2%	0.39	− 12.9%	0.18	0.57
SGLT2 inhibitors^†^	+ 34.3%	+ 33.0%	0.83	+ 34.5%	0.98	0.80
DPP-4 inhibitors^‡^	+ 9.5%	+ 5.7%	0.22	+ 4.8%	0.14	0.78
GLP-1 analogues^§^	+ 23.9%	+ 32.2%	0.068	+ 22.3%	0.73	0.031
Insulins^a^	+ 3.2%	+ 19.1%	0.001	+ 4.6%	0.76	0.003
Total	+ 7.2%	+ 8.6%	0.44	+ 4.0%	0.091	0.010

A plus sign denotes an increase; a minus sign denotes a decrease

^*^Including fixed combinations with other antidiabetic drugs

^†^*SGLT2* sodium-glucose cotransporter-2

^‡^*DPP-4* dipeptidyl peptidase-4

^§^*GLP-1* glucagon-like peptide-1

**Figure 4:**
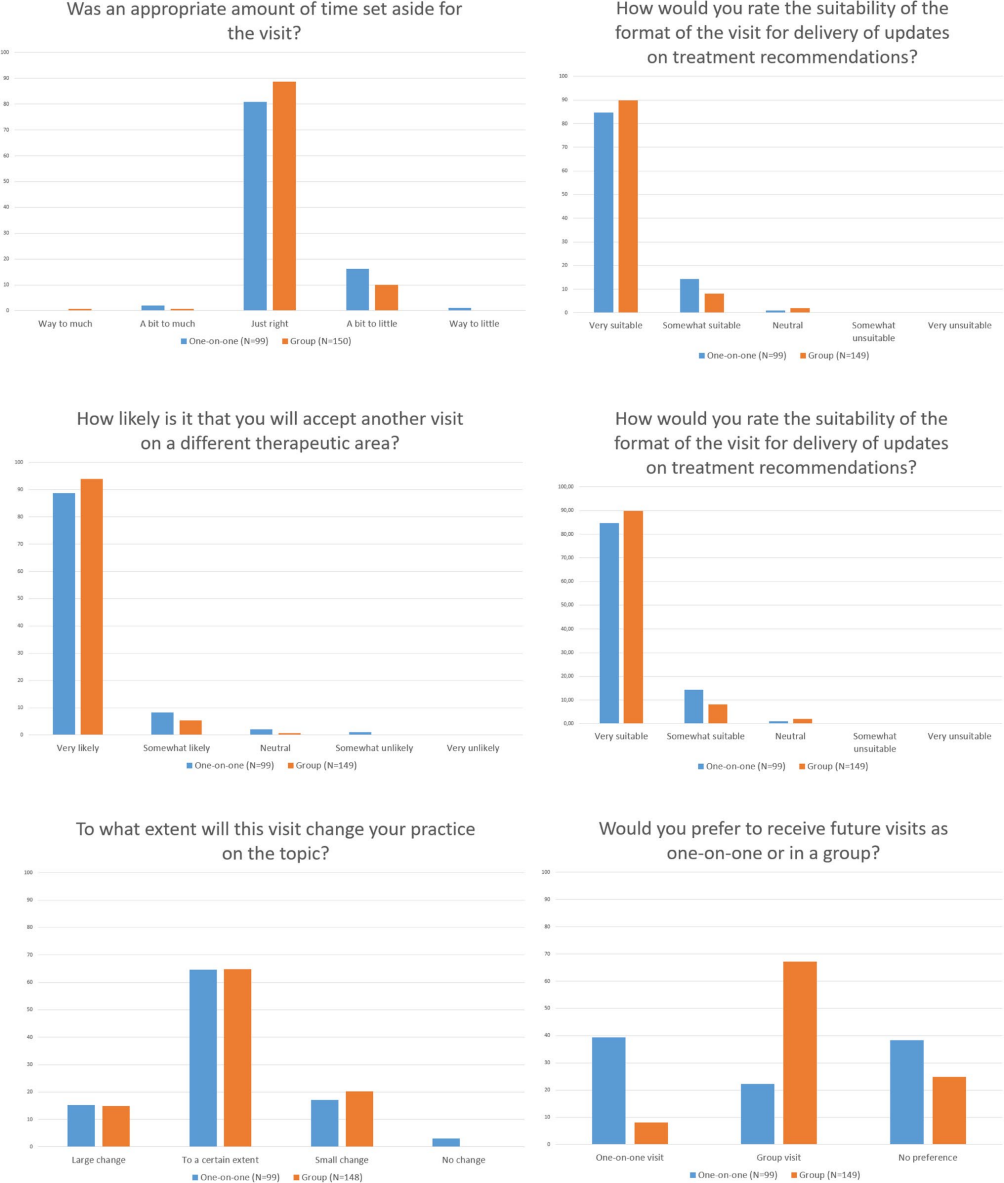
Results from the evaluation from the GPs. Numbers on the y-axis indicate percentage of answers from each intervention group.

A total of 99 (51.3%) of those receiving a one-on-one visit, and 149 (57.1%) of those receiving a group visit responded to the evaluation questionnaire. Since the evaluations were anonymous, all responses were included regardless of whether the GP was included in the analyses of prescription data. Results from the evaluations are displayed in Fig. 4.

## DISCUSSION

Both AD (one-on-one visits) and group visits increased the use of metformin compared to controls. This is consistent with the results from a previous non-randomized study from the same campaign, but in a separate population of GPs, where we found a small but statistically significant increase in prescribing of metformin among GPs receiving AD compared to a non-intervention control group.^20^ The increased use of metformin in these studies is in line with the Norwegian guidelines and recommendations at the time of intervention, and thus with the goal of the campaign.^21^ The primary endpoint in both studies was change in the use of metformin; and taken together, these studies show that both AD and group visits increased the use of metformin compared to controls, as intended. As GPs may find it difficult being updated with the latest recommendations on T2D treatment,^27^ educational outreach in the form of AD or group meetings can help GPs to keep up-to-date within a very limited timeframe.

For other T2D drugs, the differences between the two intervention groups were small, the only exception being insulin.

A previous study among Norwegian GPs performed in the period 2005–2014 found that the use of insulin for T2D was, in fact, decreasing in that period, but the authors did not suggest any cause for that finding.^28^ When discussing use of insulin with GPs during the visits, many GPs reported barriers against its use among both prescribers and patients. Patients often prefer oral treatments, and GPs can find it challenging to monitor insulin treatment compared to fixed-dose oral treatments. One possibility for the larger increase of insulin after group visits could be that group meetings to a higher degree than one-on-one meetings initiated discussions and exchange of experience with colleagues, and that peer-to-peer discussions with colleagues at the clinic made the GPs feel more confident with starting insulin treatment than a one-on-one meeting with an external academic detailer. After such discussions, the GPs also knew that they could confer with a more experienced colleague if needed.

There is also a possibility that the difference in use of insulin may be caused by factors outside our intervention. The municipalities in our study belong to seven different hospitals, and since insulin in T2D often is initiated at hospitals, changes in staff or policy at one hospital that influence the prescribing could cause a similar change at the GP level as well. There is also the possibility of random effects, but the high degree of significance in this finding makes that unlikely.

For total use of T2D drugs, the difference from the control group only reached statistical significance for group visits. Still, the trend is that group visits change prescribing slightly more than one-on-one visits. This increase is mainly due to differences in not only the use of insulin, but also the only other injectable drug group, GLP-1 analogues. Again, one possible explanation could be that group visits better facilitated discussions between colleagues sharing experiences with the use of injectable drugs.

Few previous studies have compared one-on-one and group visits with other drug groups, and the findings have been heterogeneous. In a randomized trial, Figueiras and colleagues compared the effect of 20-min one-to-one visits with 45-min group meetings to change the prescribing of NSAIDs among GPs in Spain. The quality of prescribing improved significantly in both study groups, with the largest effect among those who received a one-to-one visit.^14^ Van Eijk and colleagues found that both one-on-one and group visits decreased prescribing of anticholinergic drugs in a study from the Netherlands, without significant differences between the two interventions.^15^ Simon and colleagues studied the effect of one-on-one and group interventions to increase the use of diuretics and beta-blockers in hypertension. They found both interventions to be effective, with no significant differences between them. Interestingly, even though the effect was slightly higher after group intervention the first year, 2 years after the interventions, there was a trend suggestive of a more persistent effect after one-on-one visits.^16^

The theory behind AD is that the individual approach allows for each visit to be adjusted to the needs of the individual prescriber.^2,4,5,10,22,29^ We were not able to show a larger impact after one-on-one visits than after group meetings. This could be because the academic detailers were not skilled enough to identify each GP’s barriers and needs, or that the time set aside was too short. Evaluations from both the GPs and the academic detailers (see Supplementary File) indicate that 20 min was not sufficient for this complex subject. Another possible explanation is that group visits also had much of the same effects due to interaction between the academic detailer and the GPs, and that discussion with colleagues could have continued after the visit.

The GPs seem to prefer the type of visit they received, but to a higher degree amongst those who had group visits. None of the academic detailers preferred group meetings, and six of eight actively preferred one-on-one visits (see Supplementary File). The reason for this could be not only that they were trained specifically for one-on-one visits, but also that they felt that AD is a better way to meet the needs of every individual GP. Group meetings take almost twice as much time for each GP compared to individual visits, while being more efficient in terms of total time spent for the academic detailer.

In a study from Belgium, the acceptability of individual AD and group visits in general practice was compared. All GPs received two visits, either both individually or both in a group. Individual visits lasted on average 23 and 18 min, and group meetings 40 and 75 min, respectively. Both individual and group visits were rated positively. Although the authors do not conclude which is better, group visits scored significantly higher on two of six questions in the evaluation.^30^ Our results are in line with this study, as we also noted slightly more positive evaluations after group visits. For both studies, it is difficult to rule out that the extra times spent in group visits have contributed to the positive evaluation.

One major strength of the present study is the high number of prescribers. We were also able to follow changes in prescribing up to 1 year after intervention. An important limitation is the use of dispensing data as proxy for prescribing, meaning that it was not possible to show an immediate effect in prescribing after intervention, as dispensed prescriptions could be written up to a year before the visit. Due to the study design applied, we were also not able to study if changes in prescribing actually led to better blood glucose control or improved health outcomes for patients.

Since our study included a majority of all GPs in the region, we consider internal validity of the results as being high. It is more complicated to assess whether these findings can be generalized to other populations of GPs, although we consider that the results would be valid at least in countries where the health care system for general practice is comparable to that in Norway.

Future research should continue to investigate the different advantages of individual AD and group visits. The use of virtual visits via video-based services is also an important issue, having potential for reducing both time spent and environmental impact of visits. The effect of performing virtual visits individually or in groups still needs to be investigated.

Our work shows that short educational visits of 20–45 min will impact the prescribing of drugs for T2D; the education is given either one-on-one as AD, or in a more traditional group setting.

## Supplementary Information

Below is the link to the electronic supplementary material.

Supplementary file1 (DOCX 60 KB)
