# Supplementary material for: Academic Detailing Compared with Group Meetings to Change Drug Prescribing for Type 2 Diabetes—A Randomized Controlled Trial
Source: J Gen Intern Med. 2024 Sep 4;39(16):3243–52. doi: 10.1007/s11606-024-09014-z (PMC11618564; doi:10.1007/s11606-024-09014-z)
Supplement: Supplementary file 1 — Supplementary file1 (DOCX 60 KB) [file 11606_2024_9014_MOESM1_ESM.docx]

**Supplementary file - Evaluation from the academic detailers**

**Academic detailing compared with group meetings to change prescribing for type 2 diabetes – A randomized controlled trial**

Harald Chr. Langaas, Øyvind Salvesen, Roar Dyrkorn, Hege Salvesen Blix, Olav Spigset

**Method**

A total of 10 academic detailers participated in the visits, five experienced pharmacists and five physicians (three specialists in general practice and/or clinical pharmacology, and two residents). Training included a three-day course in the principles and methodology of AD, and additional training in the content of the campaign. All academic detailers conducted both one-on-one and group visits. All received a single, individual anonymous electronic evaluation form (Questback®) with four questions after the final visit.

**Results**

Eight (80%) of the academic detailers responded to the evaluation. Results from the evaluations are displayed in Supplementary figure 1.


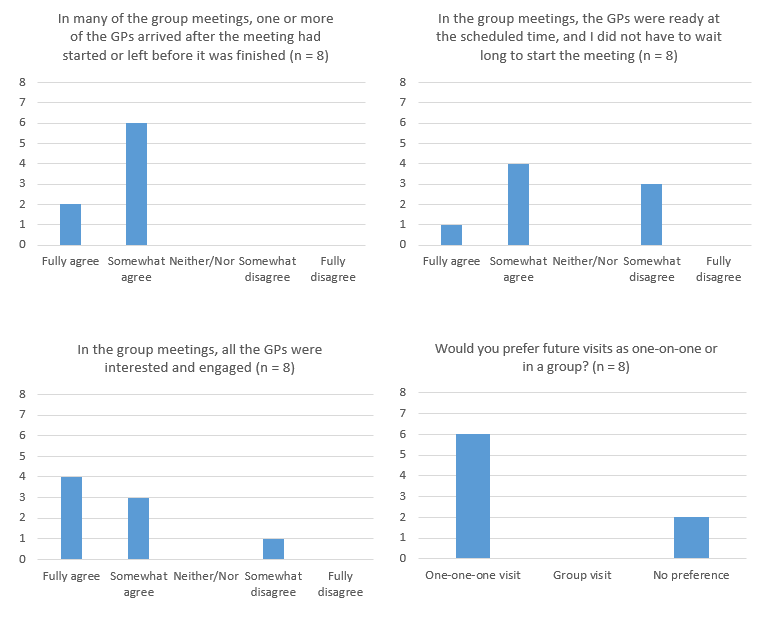


**Supplementary figure 1**. Results of the evaluation from the academic detailers. Eight of ten academic detailers responded to the evaluation. Numbers on the y axis indicate number of responses
